# Survey dataset on leadership styles and job satisfaction: The Perspective of employees of hospitality providers

**DOI:** 10.1016/j.dib.2018.06.033

**Published:** 2018-06-26

**Authors:** Ohunakin Folakemi, Adeniji A. Adenike, Oludayo A. Olumuyiwa, Adewale O. Osibanjo

**Affiliations:** Department of Business Management, College of Business and Social Sciences, Covenant University, Nigeria

**Keywords:** Idealised influence, Intellectual stimulation, Inspirational motivation, Individualised consideration, Employees’ job satisfaction

## Abstract

This study aimed at establishing the relationship between the dimensions of leadership styles and employees’ job satisfaction in hospitality industry in Nigeria. This study was prompted by reports of high labour turnover in this sector of the economy (especially in the guesthouses), because of reduction in the satisfaction of the workforce. Cross-sectional research design which is quantitative in nature, was the methodology adopted for this study to assess the trends of relationships between the constructs. Questionnaire was used as the measuring instrument, and reliability and validity test for the instrument were established using cronbach alpha, for all the variables ranging between 71% and 89%. The study population comprises 410 employees in the six selected functioning guesthouses, which also represents the study sample. Total enumeration sampling technique was adopted. Statistical Package for Social Sciences (SPSS) software package (version 22) was used for the analysis of the data. The field dataset is available to the public for more rigorous, extensive, critical and extended analysis.

**Specification Table**

**Table:** 

Subject area	Human Resource Management
More Specific Subject Area	Leadership
Type of Data	Table, figure and text file
How Data was Acquired	Through questionnaire
Data format	Raw, analysed, descriptive and inferential statistical data
Experimental Factors	– Sample consisted of employees in selected Universities’ guesthouses in southwest, Nigeria – The researcher-made questionnaire including data on demographic, data on idealised influence, inspirational motivation, intellectual stimulation, individualised consideration, contingent reward, management by exception active, management by exception passive and employees’ job satisfaction. – In this data set, the relationship between idealised influence, inspirational motivation, intellectual stimulation, individualised consideration, contingent reward, management by exception active, management by exception passive and employees’ job satisfaction had been studied
Experimental features	Leadership style in every organisation plays a significant role on the employees’ satisfaction, it also has the capabilities to make or mare organisational overall performances
Data Source Location	Southwest (Ogun State, Osun State, Oyo State and Lagos State), Nigeria
Data Accessibility	The data are available with this article

**Value of data**

• These data could assist management to discover the appropriate leadership style, which will enable the organisation to boost employees’ job satisfaction and further improve organisation׳s activities.
• The data could provide the organisation with ample information on which of the dimensions of transformational and transactional leadership styles will be the best in boosting employees’ job satisfaction.
• Generally, this data obtained from this study would be important for organizational goal and objectives achievement, gaining competitive advantage that would lead to better organizational performance.
• These data are available for more rigorous, comparative and extended analysis by other researchers.

## 1 Data

According to Table 1, four hundred and ten (410) copies of questionnaire were administered to the employees of the selected Universities guesthouses in southwest, Nigeria. Three hundred and twenty-four (324) were returned and usable, which represented 79%, while the remaining eighty-six (86) were not returned, thus representing 21% of the total questionnaire administered.

**Table 1:** Rate of response of the administered questionnaire.

Questionnaire		Number of respondents	Rate of response (%)
Administered	410		
Returned and usable		324	79
Not returned		86	21
Total	410	410	100

Socio-Demographic Profile of Respondents.

**Fig. 1:**
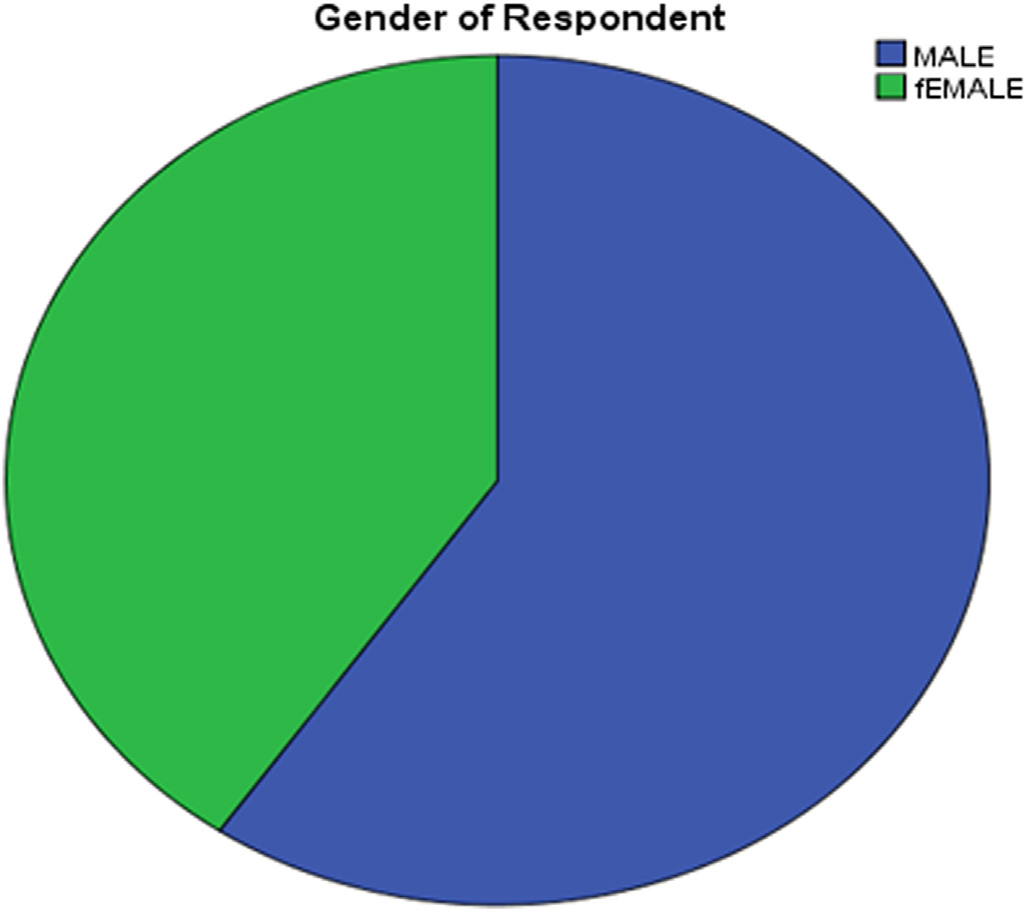
Gender of respondents.

**Fig. 2:**
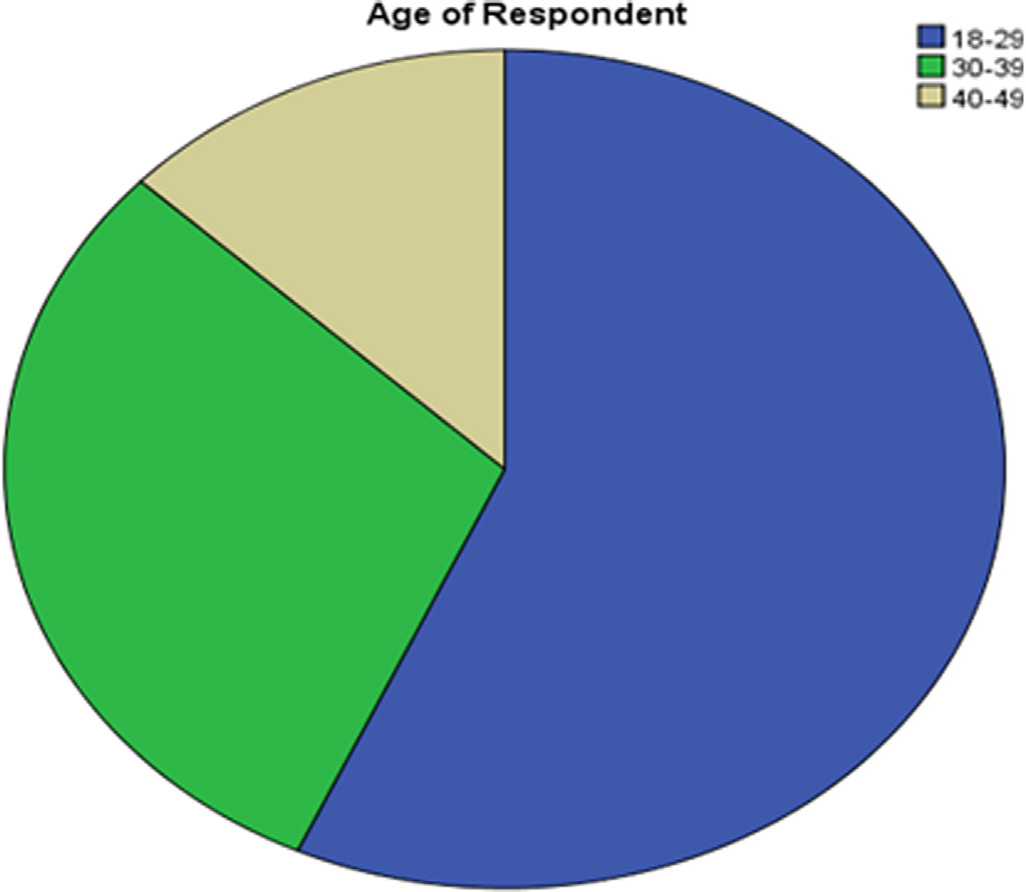
Age of respondents.

**Fig. 3:**
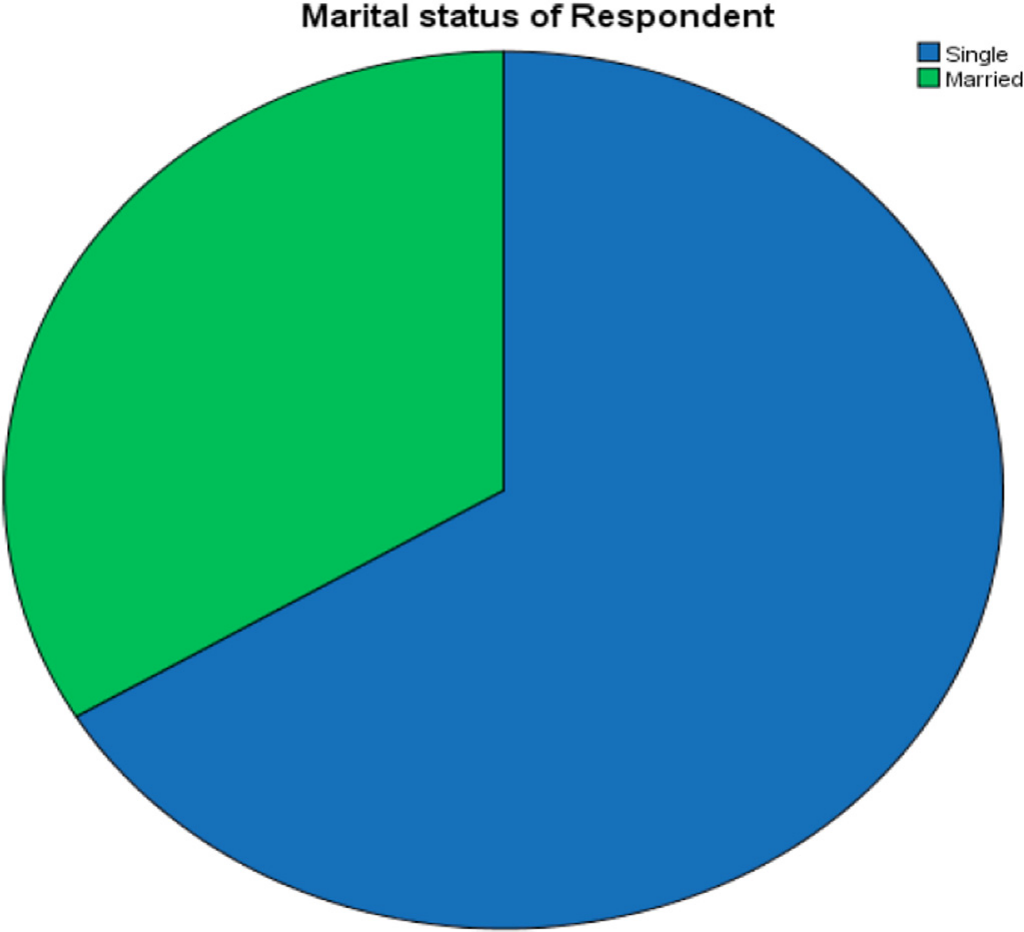
Marital status of respondents.

**Fig. 4:**
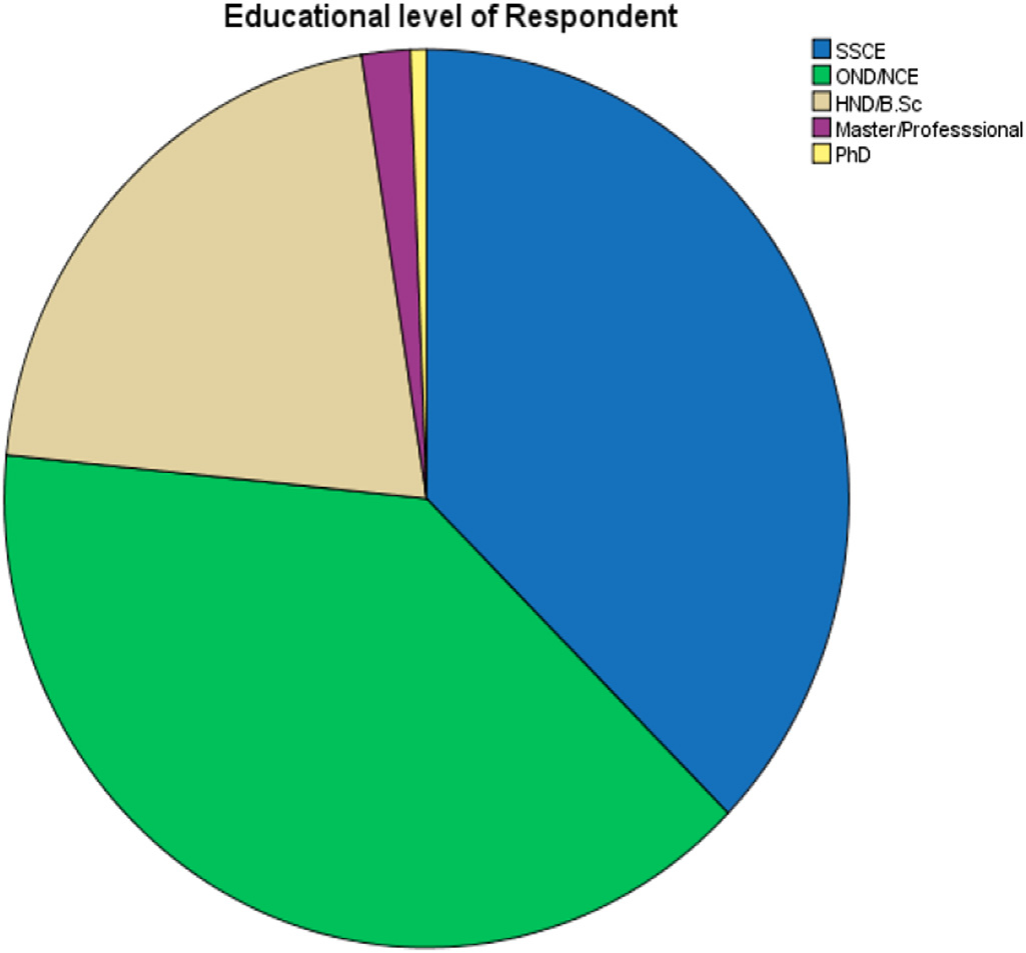
Educational level of respondents.

Based on the usable copies of questionnaire, Table 2, Table 3, Table 4, Table 5 and Fig. 1, Fig. 2, Fig. 3, Fig. 4 revealed the demographic profile of the respondents according to gender, age, marital status and educational qualification. The demographic data of the respondents revealed that 193 (59.6%) were male, while the female respondents were 131 (40.4%). Though, male respondents were more than the female respondents, but the opinion of both genders were adequately represented. Based on Table 3, ages 18–29 years were 184 (56.8%), ages between 30 and 39 were 98 (30.2%), and 42 (13.0%) were the respondents between ages 40 and 49 years. From Table 4, the singles among the respondents were 215 (66.4%), while the married were 109 (33.6%) of the total respondents. According to Table 5, 121 (37.3%) of the respondents were Senior Secondary School Certificate Examination (SSCE) holders, 127 (39.2%) of the respondents were Ordinary National Diploma (OND) and National Certificate in Education (NCE) certificate holders. Higher National Diploma (HND) and first degree holders from the University among the respondents were 68 (21.0%), Masters and Professional certificate holders among the respondents were 6 (1.9%), while 2 (0.6%) were Doctor of philosophy (Ph.D) holders among the respondents.

**Table 2:** Gender of respondent.

		Frequency	Percent	Valid percent	Cumulative percent
Valid	Male	193	59.6	59.6	59.6
	Female	131	40.4	40.4	100.0
	Total	324	100.0	100.0

**Table 3:** Age of respondent.

		Frequency	Percent	Valid percent	Cumulative percent
Valid	18–29	184	56.8	56.8	56.8
	30–39	98	30.2	30.2	87.0
	40–49	42	13.0	13.0	100.0
	Total	324	100.0	100.0

**Table 4:** Marital status of respondent.

		Frequency	Percent	Valid percent	Cumulative percent
Valid	Single	215	66.4	66.4	66.4
	Married	109	33.6	33.6	100.0
	Total	324	100.0	100.0

**Table 5:** Educational level of respondent.

		Frequency	Percent	Valid percent	Cumulative percent
Valid	SSCE	121	37.3	37.3	37.3
	OND/NCE	127	39.2	39.2	76.5
	HND/B.Sc	68	21.0	21.0	97.5
	Master/Professional	6	1.9	1.9	99.4
	PhD	2	.6	.6	100.0
	Total	324	100.0	100.0

The descriptive statistics evaluating the dimensions of transformational and transactional leadership styles and employees’ job satisfaction are as shown in Table 6, Table 7, Table 8, Table 9, Table 10, Table 11, Table 12. In line with Table 6, 146 (45.1%) of the respondents strongly agree, 73 (22.5%) agree, 35 (10.7%) partially agree, 9 (2.8%) partially disagree, 53 (16.4%) disagree, and 8 (2.5%) strongly disagree, that idealised influence of their leader will have positive effect on their job satisfaction.

**Table 6:** Descriptive statistics evaluating the effect of idealised influence on employees’ job satisfaction.

Valid	Frequency	%	Valid %	Cumulative %
Strongly disagree	146	45.1	45.1	45.1
Agree	73	22.5	22.5	67.6
Partially agree	35	10.7	10.7	78.3
Partially disagree	9	2.8	2.8	81.1
Disagree	53	16.4	16.4	97.5
Strongly disagree	8	2.5	2.5	100
Total	324	100	100

**Table 7:** Descriptive statistics assessing the effect of inspirational motivation on employees’ job satisfaction.

Valid	Frequency	%	Valid %	Cumulative %
Strongly disagree	134	41.4	41.1	41.1
Agree	79	24.4	24.4	65.5
Partially agree	43	13.3	13.3	78.8
Partially disagree	20	6.2	6.2	85.0
Disagree	42	13,0	13.1	98
Strongly disagree	6	1.9	1.9	100
Total	324	100	100

**Table 8:** Descriptive statistics evaluating the effect of intellectual stimulation on employees’ job satisfaction.

Valid	Frequency	%	Valid %	Cumulative %
Strongly disagree	133	41.1	41.1	41.1
Agree	72	22.2	22.2	63.2
Partially agree	49	15.1	15.1	78.3
Partially disagree	8	2.5	2.5	80.8
Disagree	48	14.8	14.8	95.6
Strongly disagree	14	4.3	4.3	100
Total	324	100	100

**Table 9:** Descriptive statistics evaluating the effect of individualised consideration on employees’ job satisfaction.

Valid	Frequency	%	Valid %	Cumulative %
Strongly disagree	119	36.7	36.7	36.7
Agree	86	26.5	26.5	63.2
Partially agree	53	16.4	16.4	79.6
Partially disagree	9	2.8	2.8	82.4
Disagree	47	14.5	14.5	96.9
Strongly disagree	10	3.1	3.1	100
Total	324	100	100

**Table 10:** Descriptive assessing the effect of contingent reward on employees’ job satisfaction.

Valid	Frequency	%	Valid %	Cumulative %
Strongly disagree	88	27.2	27.2	27.2
Agree	118	36.4	36.4	63.6
Partially agree	31	9.6	9.6	73.2
Partially disagree	13	4.0	4.0	77.2
Disagree	63	19.4	19.4	96.6
Strongly disagree	11	3.4	3.4	100
Total	324	100	100

**Table 11:** Descriptive statistics evaluating the relationship between management by exception active and employees’ job satisfaction.

Valid	Frequency	%	Valid %	Cumulative %
Strongly disagree	63	19.4	19.4	19.4
Agree	36	11.1	11.1	30.5
Partially agree	33	10.2	10.2	40.7
Partially disagree	58	17.9	17.9	58.6
Disagree	100	30.9	30.9	89.5
Strongly disagree	34	10.5	10.5	100
Total	324	100	100

**Table 12:** Descriptive statistics of the relationship between management by exception (passive) and employees’ job satisfaction.

Valid	Frequency	%	Valid %	Cumulative %
Strongly disagree	57	17.6	17.6	17.6
Agree	38	11.7	11.7	29.3
Partially agree	41	12.7	12.7	42.0
Partially disagree	39	12.0	12.0	54.0
Disagree	124	38.3	38.3	92.3
Strongly disagree	25	7.7	7.7	100
Total	324	100	100	

According to Table 7, 134 (41.4%) strongly agree, 79(24.4%) agree, and 43 (13.3%) partially agree that the inspirational motivation of their leader will boost their job satisfaction, whereas, 20 (6.2%) partially disagree, 42 (13.0%) disagree, and 6 (1.9%) strongly disagree that inspirational motivation of the leader will boost their job satisfaction.

In line with Table 8, 133 (41.0%) strongly agree, 72 (22.2%) agree, and 49 (15.1%) partially agree that their superior intellectual stimulation will improve their job satisfaction, while 8 (2.5%), partially disagree 48 (14.8%) disagree, and 14 (4.3%) strongly disagree that intellectual stimulation of their superior will improve their job satisfaction.

Based on Table 9, 119 (36.7%) strongly agree, 86 (26.5%) agree, and 53 (16.4%) partially agree that individualised consideration of their boss would increase their job satisfaction, whereas 9 (2.8%) partially disagree, 47 (14.5%) disagree, and 10 (3.1%) strongly disagree that individualised consideration of their boss would increase their job satisfaction.

According to Table 10, 88 (27.2%) strongly agree, 118 (36.4%) agree, and 31 (9.6%) partially agree that contingent reward from their superior will increase their job satisfaction, while 13 (9.6%) partially disagree, 63 (19.4%) disagree and 11 (3.4%) strongly disagree that contingent reward from their superior will increase their job satisfaction.

In line with Table 11, 63 (19.4%), strongly agree, 36 (11.1%) agree, and 33 (10.2%) partially agree that their leader׳s management by exception (active) will positively influence their job satisfaction, whereas 58 (17.9%) partially disagree, 100 (30.9%) disagree, and 34 (10.5%) strongly disagree that their leader׳s management by exception (passive) will positively influence their job satisfaction.

Based on Table 12, 57 (17.6%) strongly agree, 38 (11.7%) agree, and 41 (12.7%) partially agree that their superior׳s management by exception (passive) will improve their job satisfaction, while 39 (12.0%) partially disagree, 124 (38.3%) disagree, and 25 (7.7%) strongly disagree that their superior׳s management by exception (passive) will improve their job satisfaction.

## 2 The correlational relationship between the variables

The correlational relationships between idealised influence, inspirational motivation, intellectual stimulation, individualised consideration, management by exception (active), management by exception (passive) and employees’ job satisfaction are as shown in Table 13, Table 14, Table 15, Table 16, Table 17, Table 18, Table 19. The explicit forms of the equation are as follow:

Y=f(X)

**Table 13:** Correlations showing relationship between idealised influence and job satisfaction.

**Correlations**			
		IDI2	JSc2
IDI2	Pearson Correlation	1	.610**
	Sig. (2-tailed)		.000
	*N*	324	324
JSc2	Pearson Correlation	.610**	1
	Sig. (2-tailed)	.000	
	*N*	324	324

** Correlation is significant at the 0.01 level (2-tailed).

**Table 14:** Correlations showing relationship between inspirational motivation and job satisfaction.

**Correlations**			
		IM2	JSc2
IM2	Pearson Correlation	1	.570**
	Sig. (2-tailed)		.000
	*N*	324	324
JSc2	Pearson Correlation	.570**	1
	Sig. (2-tailed)	.000	
	*N*	324	324

** Correlation is significant at the 0.01 level (2-tailed).

**Table 15:** Correlation showing relationship between intellectual stimulation and job satisfaction.

**Correlations**			
		IS2	JSc2
IS2	Pearson Correlation	1	.604**
	Sig. (2-tailed)		.000
	*N*	324	324
JSc2	Pearson Correlation	.604**	1
	Sig. (2-tailed)	.000	
	*N*	324	324

** Correlation is significant at the 0.01 level (2-tailed).

**Table 16:** Correlation showing relationship between individualised consideration and job satisfaction.

**Correlations**			
		IC2	JSc2
IC2	Pearson Correlation	1	.615**
	Sig. (2-tailed)		.000
	*N*	324	324
JSc2	Pearson Correlation	.615**	1
	Sig. (2-tailed)	.000	
	*N*	324	324

** Correlation is significant at the 0.01 level (2-tailed).

**Table 17:** Correlation showing relationship between management by exception active and job satisfaction.

**Correlations**			
		MEA	JSc2
MEA	Pearson Correlation	1	.053**
	Sig. (2-tailed)		.001
	*N*	324	324
JSc2	Pearson Correlation	.053**	1
	Sig. (2-tailed)	.001	
	*N*	324	324

** Correlation is significant at the 0.01 level (2-tailed).

**Table 18:** Correlation showing relationship between management by exception (passive) and job satisfaction.

**Correlations**			
		MEP	JS
MEP	Pearson Correlation	1	− .201**
	Sig. (2-tailed)		.989
	*N*	324	324
JS	Pearson Correlation	− .201	1
	Sig. (2-tailed)	.989	
	*N*	324	324

** Correlation is significant at the 0.01 level (2-tailed).

**Table 19:** Stepwise regression coefficient showing the individual contribution of each predictor (independent variables) to the model.

**Coefficients**^a^						
Model		Unstandardized coefficients		Standardized coefficients	*t*	Sig.
		*B*	Std. Error	Beta		
1	(Constant)	1.769	.177		10.004	.000
	IC2	.529	.038	.615	13.997	.000
2	(Constant)	1.312	.187		7.026	.000
	IC2	.313	.052	.364	5.991	.000
	IDI2	.319	.056	.347	5.713	.000
3	(Constant)	1.126	.203		5.552	.000
	IC2	.207	.070	.240	2.954	.003
	IDI2	.280	.058	.305	4.830	.000
	IS2	.194	.086	.183	2.263	.024

a. Dependent Variable: JSc2.

where Y = Job Satisfaction

X = Leadership Styles (Transformational and Transactional)
X = (x_1_, x_2_, x_3_, x_4_, x_5_, x_6_, x_7_,)

where:

x_1_ = Idealised Influence of Transformational leadership style
x_2_ = Inspirational Motivation of Transformational leadership style
x_3_ = Intellectual Stimulation of Transformational Leadership style
x_4_ = Individualised Consideration of Transformational Leadership style
x_5_ = Management by Exception (Active) of Transactional Leadership style
x_6_ = Management by Exception (Passive) of Transactional Leadership style

Explicitly,

(1) $$Y=\alpha_{0}+\beta_{1}+\mu$$

(2) $$Y=\alpha_{0}+\beta_{2}+\mu$$

(3) $$Y=\alpha_{0}+\beta_{3}+\mu$$

(4) $$Y=\alpha_{0}+\beta_{4}+\mu$$

(5) $$Y=\alpha_{0}+\beta_{5}+\mu$$

(6) $$Y=\alpha_{0}+\beta_{6}+\mu$$

where:

*Y* = dependent variable (job satisfaction)
*α*_0_ = constant
*β*_1–6_ = *x*_1_–*x*_6_
*µ* = error term

Alternatively,

$$Y=\beta_{o}+\beta_{1}LDS^{j}+\mu_{i}$$

where:

*Y* = dependent variable (Job satisfaction)
*β*_o_ = constant
*β*_1_ = changes in independent variables
*LDS* = *x*_1_–*x*_6_
*j* = 1–6
*µ* = error term

## 3 Experimental design, material and method

The focus of this study was on six (6) well-functioning Universities’ guesthouses in southwest, Nigeria. The population of the employees working in the selected guesthouses is four hundred and ten (410); they were all taken as the sample because of the small size, and also for adequate representation. However, total enumeration method was the sampling technique [1]. Pen and paper questionnaire were used for gathering quantitative data. Data on demographic characteristics of the respondents were obtained, so also, data on idealised influence, inspirational motivation, intellectual stimulation, individualised consideration, contingent reward, management by exception (active), management by exception (passive) and employees’ job satisfaction were gathered. The measuring instruments were obtained from extant literature [2], [3]. The data revealed a meaningful effect of the dimensions of transformational and transactional leadership styles on employees’ job satisfaction among employees of the selected guesthouses in southwest, Nigeria. The data gathered were coded and analysed using Statistical Package for Social Sciences (SPSS) version 22. Descriptive statistics, Pearson Product Moment Correlation (PPMC) and stepwise regression were applied in the analysis.
